# Deep learning-based intelligent diagnosis and adaptive training system for university english oral proficiency

**DOI:** 10.1038/s41598-026-51608-6

**Published:** 2026-06-04

**Authors:** Rui Chai

**Affiliations:** Department of Fundamental Courses, Tianjin Vocational Institute, Tianjin, 300410 China

**Keywords:** Oral proficiency assessment, Deep learning, Adaptive learning system, Knowledge tracing, Reinforcement learning, Computer-assisted language learning, Computational biology and bioinformatics, Mathematics and computing

## Abstract

The development of oral English proficiency among university students remains constrained by limitations in traditional assessment approaches and the scarcity of personalized training resources. This study proposes an intelligent diagnosis and adaptive training system integrating deep learning technologies with multidimensional oral proficiency assessment theory. The diagnostic component employs a multi-task learning framework combining CNN-LSTM architectures with attention-based multimodal fusion to simultaneously evaluate pronunciation accuracy, fluency, vocabulary-grammar complexity, and content coherence from continuous speech samples. The adaptive training component utilizes reinforcement learning to optimize personalized practice recommendation sequences based on dynamically tracked learner competence states. Experimental validation on a corpus of 4,374 recordings from 486 university students, using a strict speaker-independent data partitioning protocol, demonstrates that the diagnostic model achieves correlation coefficients of 0.887, 0.862, 0.824, and 0.793 with human expert ratings across the four assessment dimensions respectively. Controlled training experiments over eight weeks reveal that learners receiving reinforcement learning-optimized content sequencing attain normalized learning gains approximately 2.2 times higher than those following fixed curricula (Cohen’s d = 1.34, 95% CI [0.87, 1.81]), while maintaining stronger engagement throughout the training period. These findings provide preliminary evidence supporting the potential of the proposed framework for scalable, individualized oral English instruction in higher education contexts, though further validation across broader populations and longer time periods is warranted.

## Introduction

Teaching spoken English at the university level has long run into obstacles that ordinary pedagogical fixes rarely resolve. Curricula have been reworked, methods renewed, and yet many Chinese university students still struggle to hold their own in genuine spoken exchanges^1^. The cause is not simply too few contact hours or scarce teaching materials. What we are looking at, I would argue, is a deeper tangle: assessment systems that reward written accuracy far more than spoken fluency, classrooms where real interaction is hard to come by, and feedback that arrives too late to shape the skill while it is still forming^2^. On top of this, employers increasingly expect graduates who can actually communicate across cultures in professional settings—a pressure that has pushed schools to rethink how oral instruction ought to be organised.

Recent progress in computational linguistics and machine learning has begun to open up genuinely new routes around these pedagogical impasses. Speech recognition, once hobbled by tight vocabulary ceilings and inflexible pronunciation templates, now rivals human transcription across a broad range of acoustic settings and speaker profiles^3^. Natural language processing has travelled some distance beyond surface syntactic parsing as well: modern systems pick up on semantic coherence, pragmatic fit, and the kinds of discourse patterns that separate communicatively effective speech from merely fluent output^4^. Together, these advances have made it plausible—no longer merely aspirational—to build intelligent language-learning platforms that scale to large cohorts while still responding to individual performance trajectories^5^.

Yet when existing computer-assisted oral assessment and training platforms are examined against the genuinely multidimensional character of spoken proficiency, notable gaps come into view. Most commercial products still gauge pronunciation by matching segments against native-speaker templates and largely ignore suprasegmental cues—intonation shape, stress placement, rhythmic organisation—that actually do the heavy lifting in communication^6^. Diagnostic outputs tend to collapse an intricate speaking performance into a single score or a crude category, leaving the learner with little idea of what, specifically, needs work^7^. And the feedback loop itself is often too slow: results appear only after the whole task has ended, which rules out the kind of immediate corrective input that procedural skill acquisition really depends on^8^.

The personalisation problem is, if anything, more troubling still. Intelligent tutoring systems for oral language typically march learners through a fixed progression, with little regard for differences in pace, background knowledge, or the ways in which an individual actually prefers to practise^9^. Even where some form of adaptation is built in, it usually reacts to surface performance rather than to the deeper competence profile underneath. The effect is that interventions treat symptoms, not causes. Such a uniform approach also runs against a settled finding in second-language acquisition research: learners build oral proficiency along genuinely heterogeneous developmental paths, and there is no single route that fits all of them^10^.

Deep learning architectures look like a genuinely promising response to the shortcomings set out above, chiefly because they support hierarchical feature representation and end-to-end optimisation in one stroke. Convolutional and recurrent networks can pick up acoustic-phonetic patterns across multiple temporal scales on their own, without anyone hand-crafting the feature set beforehand^11^. Attention mechanisms, meanwhile, let models decide how much weight to give to different parts of an utterance when estimating proficiency. Perhaps most importantly—and this matters a great deal for educational work—transfer learning makes it possible to borrow what has been learned from massive speech corpora and carry it into settings where domain-specific training data are scarce^12^.

Three interlocking research objectives guide what follows, each targeting one of the gaps just described. The first is to build a multidimensional diagnostic framework that can, from a continuous speech sample, judge pronunciation accuracy, lexical-grammatical complexity, discourse coherence, and the deployment of communicative strategies all at once. The second is to construct an adaptive training model—one that recommends practice calibrated to the learner’s current competence profile and that responds, in real time, to fluctuations in performance. The third is to put the whole system to the test empirically with university English learners drawn from a range of proficiency levels.

Three innovation areas, in particular, set this work apart from earlier efforts. On the feature fusion side, a hierarchical attention architecture brings acoustic, linguistic, and discourse-level representations together dynamically, while keeping the fusion interpretable enough to drive diagnostic feedback. For the diagnostic model itself, a multi-task learning framework jointly optimises dimension-specific scoring and overall proficiency estimation, turning the interdependencies among competence facets into a source of predictive robustness rather than noise. And on the adaptive-strategy side, a reinforcement learning formulation treats training-sequence generation as a sequential decision problem, aiming at long-term learning gains instead of immediate performance bumps.

Taken together, these contributions matter for both theory and practice in foreign language education. On the theoretical side, the work extends current models of oral proficiency assessment by translating multidimensional competence constructs into computationally tractable representations. The adaptive training framework, for its part, embeds principles drawn from cognitive load theory and skill-acquisition research inside an algorithmically implemented instructional design. Practically speaking, a system of this kind could widen access to individualised oral coaching—hitherto something only affordable through human tutoring—while also generating diagnostic insights that feed back into curriculum design and teacher preparation.

### Theoretical foundations and key technologies

#### Theoretical framework for university english oral proficiency assessment

How we conceptualise oral language proficiency has shifted substantially since the earliest models, which tended to treat speaking as a single, unitary ability that could be judged holistically. Contemporary assessment theory, by contrast, treats spoken competence as inherently multidimensional: a set of distinct but interrelated component skills whose joint operation underpins communicative effectiveness^13^. The implications for diagnostic system design are considerable. If feedback is to be genuinely useful, overall performance has to be broken down into constituent dimensions that both learners and instructors can actually target through focused intervention.

Communicative competence theory, first set out by Hymes and later elaborated in Canale and Swain’s influential framework, identifies four components that sit beneath effective language use: grammatical competence for mastery of the linguistic code, sociolinguistic competence for contextual appropriateness, discourse competence for coherent text construction, and strategic competence for repairing communication when it breaks down^14^. Bachman’s communicative language ability model pushed these ideas further, drawing a line between organisational and pragmatic knowledge and giving explicit weight to the metacognitive strategies that mediate how knowledge actually gets deployed in authentic communicative settings^15^. It is on this conceptual scaffolding that any operationalised assessment criteria need to be built.

Quantifying pronunciation accuracy ordinarily means leaning on acoustic-phonetic measures that compare learner productions against native-speaker reference patterns. Segmental accuracy can be captured through phoneme-error-rate metrics, while prosodic adequacy calls for extraction of fundamental frequency contours, duration ratios, and intensity variations. Fluency assessment, in turn, combines temporal measures—speech rate computed as syllables per unit time—with pause phenomena, which can be expressed as:

1 $$\:{F}_{score}=\frac{{N}_{syllables}}{{T}_{total}-{T}_{silent}}\times(1-\frac{{N}_{repairs}}{{N}_{utterances}})$$

where $\:{T}_{silent}$ denotes cumulative pause duration and $\:{N}_{repairs}$ represents disfluency instances^16^.

For lexical richness, the standard toolkit includes variants of the type-token ratio alongside more sophisticated diversity indices that correct for text-length effects. Grammatical complexity is typically approached by combining measures of subordination density, clause length, and syntactic variety^17^. Pragmatic appropriateness is trickier—it resists any tidy quantification—but reasonable approximations can be had from discourse-act recognition and register-matching algorithms trained on contextually annotated corpora^18^. The integrated multidimensional proficiency score adopted here pulls these component evaluations together through a weighted combination:

2 $$\:{P}_{oral}=\sum_{i=1}^{n}{w}_{i}\cdot{D}_{i}\left(x\right)$$

where $\:{D}_{i}\left(x\right)$ represents dimension-specific diagnostic scores and $\:{w}_{i}$ denotes empirically derived importance weights reflecting each dimension’s contribution to overall communicative success. This formulation enables both holistic proficiency estimation and targeted diagnostic feedback addressing specific competence deficits.

#### Deep learning technologies for speech processing

Applying deep neural networks to speech signals has reshaped what automatic speech recognition and spoken language understanding can do. Earlier approaches leaned heavily on handcrafted acoustic features paired with statistical models that had only limited capacity for capturing the intricate spectro-temporal patterns woven into continuous speech^19^. Deep architectures take a different route: they learn hierarchical representations directly from raw or minimally processed audio, moving step by step—through successive nonlinear transformations—from low-level acoustic properties up to high-level linguistic units.

Convolutional networks prove especially well-suited to acoustic feature extraction, thanks to their knack for detecting local patterns that remain invariant under temporal shifts. When they are run over spectrogram representations of speech, their convolutional layers pick out formant structures, spectral transitions, and phonetically relevant frequency modulations using learnable filter banks. For a given input feature map * X* and kernel * W* follows the standard formulation:

3 $$\:{Y}_{i,j}=\sum_{m}^{}\sum_{n}^{}{X}_{i+m,j+n}\cdot{W}_{m,n}+b$$

where the bias term * b* provides an additional learnable parameter^20^. Pooling operations subsequent to convolution reduce dimensionality while preserving salient acoustic information.

Recurrent networks tackle the inherently sequential nature of speech by keeping hidden-state representations alive across time steps. Plain RNNs, however, run into the well-known vanishing-gradient problem, which hampers the learning of long-range temporal dependencies. Long Short-Term Memory networks get around this through gating mechanisms that regulate how information flows in and out of the cell. The cell-state update takes the following form:

4 $$\:{c}_{t}={f}_{t}\odot\:{c}_{t-1}+{i}_{t}\odot\:{\stackrel{\sim}{c}}_{t}$$

where $\:{f}_{t}$ and $\:{i}_{t}$ represent forget and input gate activations respectively, and $\:\odot\:$ denotes element-wise multiplication^21^. This architecture has proven remarkably successful for acoustic modeling tasks requiring sensitivity to extended temporal contexts.

The Transformer architecture, first developed for machine translation, has gradually displaced recurrent approaches in a good deal of speech processing work^22^. Self-attention lets the model describe the relationship between any two positions in an input sequence directly, sidestepping the sequential-processing bottlenecks that slow RNNs down. The attention computation itself can be written as:

5 $$\:Attention(Q,K,V)=softmax\left(\frac{Q{K}^{T}}{\sqrt[]{{d}_{k}}}\right)V$$

where * Q*, * K*, and * V* denote query, key, and value matrices derived from input representations, and $\:{d}_{k}$ provides a scaling factor preventing gradient instability^23^. Multi-head attention extends this mechanism by projecting inputs into multiple subspaces, capturing diverse relational patterns simultaneously.

End-to-end speech recognition models mark the natural culmination of these architectural moves: they map acoustic input straight to text output, without any intermediate phonetic representation. Connectionist Temporal Classification loss makes it possible to train directly on unsegmented speech data, by marginalising over all valid alignments:

6 $$\:{\mathcal{L}}_{CTC}=-\mathrm{l}\mathrm{n}\sum_{\pi\:\in\:{\mathcal{B}}^{-1}\left(y\right)}^{}\prod_{t=1}^{T}p\left({\pi\:}_{t}\right|x)$$

where Β⁻¹(y) encompasses all label sequences collapsing to target transcription y^24^. More recent encoder-decoder frameworks with attention-based alignment have achieved remarkable recognition accuracy, providing the technological foundation upon which diagnostic oral assessment systems can be constructed^25^.

#### Adaptive learning and knowledge tracing models

Adaptive learning systems rest on a pedagogical philosophy that is genuinely different from traditional instruction, which hands the same content to every student in the same order. The premise is straightforward enough: learning is at its most effective when the difficulty of the task, its pacing, and the selection of content all adjust dynamically to each student’s evolving competence state^26^. Realising this vision, though, is not trivial—it requires computational mechanisms that can infer latent knowledge states from observable performance data, and it is precisely this challenge that has given rise to the field of knowledge tracing.

Early work on knowledge tracing took a Bayesian Knowledge Tracing approach grounded in Hidden Markov Models. In these formulations, learner knowledge was represented as a binary latent variable that transitioned between unmastered and mastered states under learning and forgetting probabilities. The probability of a correct response given the knowledge state comes out as:

7 $$\:P\left(correc{t}_{t}\right|{K}_{t}=1)=1-s$$

where * s* denotes the slip parameter capturing careless errors despite mastery^27^. While conceptually elegant, such models struggled with the independence assumptions and limited representational capacity that constrained their predictive accuracy.

Deep Knowledge Tracing changed the field in a fairly dramatic way by replacing explicit probabilistic graphical models with recurrent neural networks capable of learning complex temporal patterns directly from interaction sequences^28^. Hidden-state evolution follows standard LSTM dynamics, and the output layer produces performance predictions across the knowledge-component space. The predictive gains over traditional methods were substantial, although the approach did raise concerns about interpretability—exactly what the network ends up learning is harder to characterise—and has kept researchers busy ever since.

A more recent development—Graph Neural Network-based knowledge tracing—addresses the relational structure that holds among knowledge components. By encoding prerequisite relationships and conceptual similarities as graph edges, these models propagate information across connected nodes and end up with richer knowledge representations as a result^29^. The message-passing mechanism can be written as:

8 $$\:{h}_{v}^{(l+1)}=\sigma\:({W}^{\left(l\right)}\sum_{u\in N\left(v\right)}^{}\frac{{h}_{u}^{\left(l\right)}}{\left|N\right(v\left)\right|}+{B}^{\left(l\right)}{h}_{v}^{\left(l\right)})$$

where $\:N\left(v\right)$ denotes the neighborhood of node * v* and successive layers aggregate increasingly distant contextual information^30^.

Personalised recommendation algorithms complement knowledge tracing by picking appropriate learning activities out of available content repositories. Collaborative filtering finds similar learners whose successful trajectories might then inform recommendations for a target student. Content-based approaches, in contrast, try to match activity characteristics to diagnosed weakness areas. Hybrid systems that bring both paradigms together have shown particular promise for language learning, where the sheer diversity of available content and the heterogeneity of learners make recommendation unusually hard^31^.

Reinforcement learning is arguably the most principled framework on offer for optimising adaptive training strategies. Once instructional sequencing is formulated as a Markov Decision Process—with learner knowledge states as environment states and content selection as actions—it becomes possible to learn policies that maximise long-term educational outcomes. The optimal policy satisfies the Bellman equation:

9 $$\:{Q}^{\mathrm{*}}(s,a)=R(s,a)+\gamma\:\sum_{{s}^{{\prime\:}}}^{}P\left({s}^{{\prime\:}}\right|s,a\left){\mathrm{m}\mathrm{a}\mathrm{x}}_{{a}^{{\prime\:}}}{Q}^{\mathrm{*}}\right({s}^{{\prime\:}},{a}^{{\prime\:}})$$

where future rewards are discounted by factor $\:\gamma\:$ and the state transition probabilities capture learning dynamics^32^. Such formulations naturally handle the delayed reward structure characteristic of educational settings, where immediate performance gains may not reflect durable learning.

### Intelligent diagnosis and adaptive training model construction

#### Overall system architecture design

The proposed system for intelligent oral proficiency diagnosis and adaptive training is organised around a hierarchical architecture that separates concerns while keeping information flowing cleanly between processing stages. The design philosophy behind this choice is unashamedly practical: modularity, maintainability, and the fact that different system components place quite different demands on compute resources^33^. A monolithic implementation would have made debugging and incremental improvement far harder than they need to be. Instead, we adopt a layered approach in which each stratum does a well-defined job and communicates with the others through standardised interfaces.

**Fig. 1:**
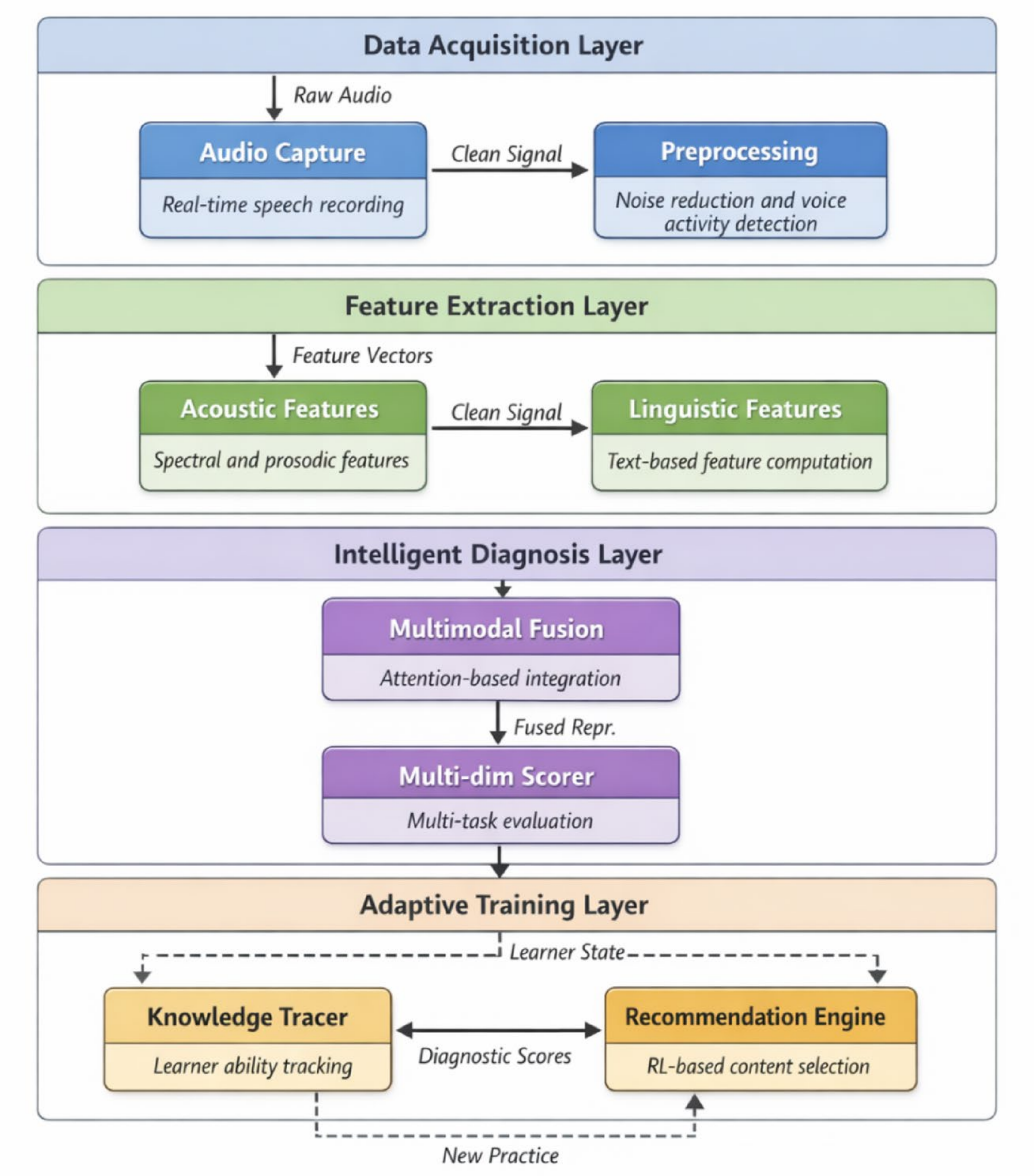
Overall system architecture of the intelligent oral diagnosis and adaptive training system, depicting the four-layer hierarchy and inter-layer data interfaces.

The architecture comprises four principal layers, each addressing specific aspects of the diagnostic and training pipeline. Figure 1 illustrates the overall system architecture depicting these layers and their interconnections.

As Fig. 1 shows, the Data Acquisition Layer acts as the system’s sensory interface: it handles audio capture, runs noise-reduction preprocessing, and applies voice activity detection to break continuous recordings into analysable speech segments. The Feature Extraction Layer then transforms raw acoustic signals into multi-granularity representations suitable for downstream analysis—from frame-level spectral features through phoneme-level embeddings to utterance-level semantic encodings^34^. The Intelligent Diagnosis Layer houses the core neural network models that evaluate oral performance across the multidimensional assessment framework established in Sect.("Theoretical framework for university english oral proficiency assessment"). Finally, the Adaptive Training Layer consumes diagnostic outputs to generate personalized practice recommendations and dynamically adjust difficulty parameters.

Table 1 summarizes the functional modules within each architectural layer along with their primary responsibilities and key technologies employed.

**Table 1:** System functional module division.

Layer	Module	Primary Function	Key Technologies
Data Acquisition	Audio Capture Module	Real-time speech recording and streaming	WebRTC, WAV encoding
Data Acquisition	Preprocessing Module	Noise reduction and voice activity detection	Spectral subtraction, VAD algorithms
Feature Extraction	Acoustic Feature Module	Spectral and prosodic feature extraction	MFCC, Mel-spectrogram, F0 analysis
Feature Extraction	Linguistic Feature Module	Text-based feature computation	Pre-trained language models, dependency parsing
Intelligent Diagnosis	Multi-dimensional Scorer	Proficiency dimension evaluation	Multi-task CNN-LSTM, attention mechanisms
Adaptive Training	Recommendation Engine	Personalized content selection	Reinforcement learning, knowledge tracing

The data flow that ties these layers together is predominantly feed-forward during inference, though feedback loops do exist—they allow continuous model refinement as learner interaction data accumulate^35^. Raw audio streams enter through the acquisition interface, undergo preprocessing to strip out environmental noise, and then pass into parallel feature-extraction pipelines targeting the acoustic and linguistic representation spaces respectively. These heterogeneous feature vectors converge at the diagnosis layer, where fusion mechanisms combine the information streams before dimension-specific scoring. From there, the diagnostic results propagate onward to the training layer, where the recommendation algorithms pick out appropriate practice activities.

**Fig. 2:**
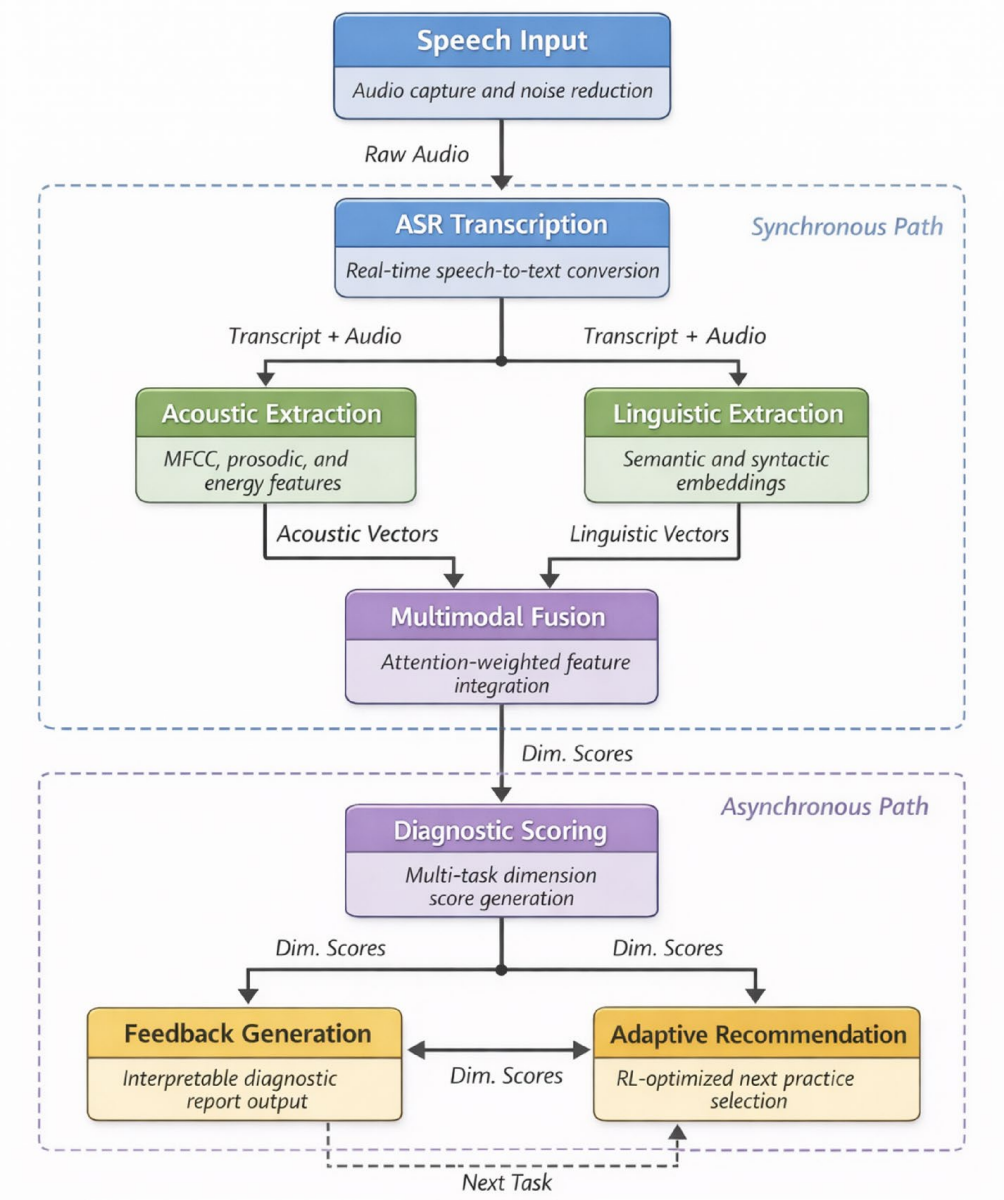
Presents the core processing flow that traces a single oral practice attempt from initial recording through diagnostic feedback generation and subsequent training recommendation.

Figure 2. Core processing flow illustrating the sequential pipeline for a single oral practice attempt, from speech input through diagnostic scoring to adaptive training recommendation, with synchronous and asynchronous processing stages distinguished.

While Fig. 1 provides a static, architectural overview of the system’s four layers and their logical connections, Fig. 2 traces the dynamic, temporal sequence of operations that unfold when a learner submits a single oral practice attempt. As demonstrated in Fig. 2, the processing pipeline incorporates both synchronous and asynchronous operations. Time-critical components including speech recognition and real-time feedback generation execute synchronously to minimize perceptible latency, while computationally intensive operations such as detailed diagnostic analysis and learner model updates proceed asynchronously without blocking user interaction^36^.

The technical implementation rests on established frameworks optimised for deep learning inference. PyTorch is the primary neural-network development platform, with TorchScript compilation handling deployment-side optimisation. The speech processing pipeline draws on the Kaldi toolkit for acoustic feature extraction and, for language-model integration, HuggingFace Transformers. Redis provides the caching infrastructure for frequently accessed learner profiles; PostgreSQL handles persistent storage of interaction logs and diagnostic histories. This technology stack balances development efficiency against runtime performance requirements characteristic of real-time educational applications.

#### Multi-dimensional oral feature extraction and fusion network

Effective oral proficiency diagnosis depends on feature representations that can do justice to the multifaceted nature of spoken language production. A speaker’s communicative competence shows up all at once—in segmental pronunciation accuracy, in suprasegmental prosodic patterns, in lexical choice, syntactic organisation, and discourse coherence. These dimensions each call for their own extraction methodology, yet they need to be brought together for holistic assessment to work. What follows lays out our approach to building parallel feature-extraction pathways and the fusion mechanism that stitches their outputs back together.

The acoustic feature extraction module works directly on preprocessed audio signals to produce low-level spectral representations. Mel-frequency cepstral coefficients remain the foundational descriptors here, and they are computed along a standard pipeline: framing, windowing, Fourier transformation, mel-filterbank application, and a final discrete cosine transformation^37^. For a given frame, the MFCC computation proceeds as:

10 $$\:{c}_{n}=\sum_{m=1}^{M}log\left({S}_{m}\right)\cdot cos\left[\frac{\pi\:n(m-0.5)}{M}\right]$$

where $\:{S}_{m}$ represents the energy in the * m*-th mel-filterbank channel and * M* denotes the total number of filters. Beyond static coefficients, we extract delta and delta-delta features capturing temporal dynamics. Fundamental frequency estimation employs autocorrelation-based pitch tracking, with the F0 contour providing essential information about intonation patterns. Frame-level energy, calculated as:

11 $$\:E=10\cdot {log}_{10}(\sum_{n=1}^{N}{x}^{2}\left[n\right]+\epsilon)$$

where *ε* prevents logarithm of zero, supplements the feature set with intensity variation information relevant to stress pattern analysis^38^.

Table 2 summarizes the comprehensive multi-dimensional feature system adopted in this research, organizing extracted features by category and indicating their diagnostic relevance.

**Table 2:** Multi-dimensional oral feature system.

Feature Category	Specific Features	Extraction Method	Dimensionality	Diagnostic Target
Spectral	MFCC, delta, delta-delta	DCT on mel-spectrogram	39 per frame	Pronunciation accuracy
Prosodic-Pitch	F0 mean, range, slope	Autocorrelation tracking	12 per utterance	Intonation patterns
Prosodic-Rhythm	Speech rate, pause ratio	Temporal segmentation	8 per utterance	Fluency assessment
Prosodic-Energy	Intensity contour, dynamics	Frame-level computation	6 per utterance	Stress placement
Lexical	Word embeddings, diversity	Pre-trained LM encoding	768 per utterance	Vocabulary richness
Syntactic	Dependency representations	Parser-based extraction	256 per utterance	Grammatical complexity
Semantic	Contextual embeddings	BERT-based encoding	768 per utterance	Content coherence
Discourse	Cohesion markers, structure	Sequence-level analysis	128 per utterance	Discourse organization

To clarify the operationalization of communicative strategy use as an assessment dimension, we define it through three computable indicators derived from discourse-level analysis: (1) communication breakdown repair frequency, measured as the count of self-corrections, rephrasing attempts, and circumlocution instances per minute of speech; (2) discourse management markers, quantified through the frequency of cohesive devices (e.g., connectives, referential expressions) normalized by utterance length; and (3) interactional strategy deployment, captured via turn-taking cues and hedging expressions identified through pattern matching against a curated lexicon of 127 English pragmatic markers. These indicators are encoded as a 128-dimensional vector within the discourse feature category shown in Table 2 and are jointly learned during multi-task training.

The semantic feature extraction module processes transcribed speech using pre-trained transformer-based language models. We use a fine-tuned BERT architecture that produces contextualised word representations, which are then pooled up to utterance-level embeddings^39^. The pooling operation itself combines the [CLS] token representation with attention-weighted averaging:

12 $$\:{h}_{utt}=\alpha\:\cdot{h}_{\left[CLS\right]}+(1-\alpha\:)\cdot \sum_{i=1}^{L}{a}_{i}\cdot{h}_{i}$$

where attention weights $\:{a}_{i}$ are learned during task-specific fine-tuning and $\:\alpha\:$ balances the contribution of global versus local representations.

The prosodic feature analysis module zooms in on the suprasegmental characteristics that separate fluent from disfluent speech. Rhythm metrics derive from syllable-duration distributions, with the Pairwise Variability Index doing most of the work in quantifying temporal regularity:

13 $$\:PVI=\frac{100}{n-1}\sum_{k=1}^{n-1}\left|\frac{{d}_{k}-{d}_{k+1}}{({d}_{k}+{d}_{k+1})/2}\right|$$

where $\:{d}_{k}$ represents the duration of the * k*-th vocalic interval^40^. Pause analysis distinguishes filled from silent hesitations and categorizes pauses by position relative to syntactic boundaries.

The multimodal feature fusion mechanism addresses a genuinely thorny problem: how to integrate heterogeneous representations that differ not just in dimensionality but in their statistical properties. Simple concatenation, which would treat all features as equally informative regardless of context, seemed to us far too blunt an instrument. Instead, we adopt an attention-based fusion strategy in which the fused representation emerges through:

14 $$\:{F}_{fused}=\sum_{j=1}^{K}{\beta\:}_{j}\cdot{W}_{j}\cdot{f}_{j}$$

where $\:{f}_{j}$ denotes the * j*-th modality feature vector, $\:{W}_{j}$ provides modality-specific projection matrices, and attention weights $\:{\beta\:}_{j}$ are dynamically computed based on input characteristics^41^. This mechanism enables the diagnostic model to emphasize different feature categories depending on the specific assessment dimension under evaluation—prioritizing acoustic features for pronunciation scoring while attending more heavily to semantic representations when evaluating content coherence.

#### Intelligent diagnosis model and adaptive training strategy

The diagnostic model architecture sits on top of the fused feature representations described above, turning these inputs into multidimensional proficiency assessments via a carefully laid-out neural network structure. One design decision stood out as critical: should separate models be trained for each assessment dimension, or should the system pursue joint optimisation through shared representations? We went with the latter, reasoning that pronunciation accuracy, fluency, and content quality—conceptually distinct though they are—share underlying dependencies that multi-task learning can put to work^42^.

The multi-task learning framework uses a shared encoder feeding into dimension-specific prediction heads. The shared encoder, implemented as a bidirectional LSTM with self-attention, processes the fused feature sequence to produce contextualised hidden representations. Each task-specific head then computes its own dimension scores through dedicated fully-connected layers. The joint training objective combines the individual losses:

15 $$\:{\mathcal{L}}_{total}=\sum_{d=1}^{D}{\lambda\:}_{d}{\mathcal{L}}_{d}+\mu{\mathcal{L}}_{reg}$$

where $\:{\mathcal{L}}_{d}$ represents the loss for dimension * d*, $\:{\lambda\:}_{d}$ controls task importance weighting, and $\:{\mathcal{L}}_{reg}$ provides regularization against overfitting^43^. This formulation enables knowledge transfer across related assessment dimensions while accommodating their distinct scoring characteristics.

The dimension-specific loss weights in the multi-task objective were determined through uncertainty-based weighting following the homoscedastic uncertainty approach^43^. Specifically, each task weight is parameterized as the inverse of a learned observation noise scalar, allowing the optimization process itself to calibrate relative task importance rather than relying on manual tuning. We initialized all weights equally and verified through grid search that the learned weights (pronunciation: 0.28, fluency: 0.26, vocabulary-grammar: 0.24, content: 0.22) aligned with the relative annotation reliability across dimensions, where higher-reliability dimensions received slightly larger weights.

Generating diagnostic feedback that is actually interpretable—not merely numerical scoring—is essential for any educational application. We handle this through attention-based explanation mechanisms that flag the speech segments most influential for each dimension’s evaluation. Attention weights over input frames are computed via:

16 $$\:{\alpha\:}_{t}^{\left(d\right)}=\frac{exp\left({e}_{t}^{\left(d\right)}\right)}{\sum_{k=1}^{T}exp\left({e}_{k}^{\left(d\right)}\right)}$$

where the energy function $\:{e}_{t}^{\left(d\right)}={v}_{d}^{T}\cdot\:tanh({W}_{d}{h}_{t}+{b}_{d})$ produces dimension-specific relevance scores^44^. By extracting time intervals receiving maximal attention weights, the system can pinpoint specific pronunciation errors, hesitation locations, or grammatically problematic segments for targeted feedback.

Modelling the learner’s ability state calls for tracking proficiency as it evolves across practice sessions. We extend deep knowledge tracing by folding the multidimensional assessment structure directly into the hidden-state representation. The learner state update takes the following form:

17 $$\:{s}_{t+1}=GRU({s}_{t},[{d}_{t};{r}_{t};\varDelta\:t\left]\right)$$

where $\:{d}_{t}$ encodes the diagnostic vector from interaction * t*, $\:{r}_{t}$ represents the practiced content embedding, and $\:\varDelta\:t$ captures temporal spacing effects relevant to memory consolidation^45^. This continuous state representation enables fine-grained ability estimation beyond binary mastery assumptions.

We treat adaptive training strategy optimisation as a sequential decision problem, which makes it a natural candidate for reinforcement learning. The state space is defined through the current learner ability vector; the action space, through available practice activities along with their associated difficulty and focus dimensions; and the reward signal, through a composite function that combines diagnostic score improvement (weighted 0.6), task completion rate (weighted 0.2), and time-on-task efficiency (weighted 0.2)—so both direct proficiency gains and indirect engagement signals enter the picture. The policy network then outputs action selection probabilities:

18 $$\:{\pi}_{\theta}\left(a\right|s)=softmax({W}_{\pi}\cdot ReLU({W}_{s}\cdot s+{b}_{s})+{b}_{\pi})$$

Training employs the Proximal Policy Optimization algorithm, which constrains policy updates to prevent destabilizing large changes:

19 $$\:{\mathcal{L}}^{PPO}\left(\theta\:\right)=\mathbb{E}\left[\mathrm{m}\mathrm{i}\mathrm{n}\right({r}_{t}\left(\theta\:\right)\hat {{A}}_{t},clip\left({r}_{t}\right(\theta\:),1-\epsilon,1+\epsilon)\hat {{A}}_{t}\left)\right]$$

where r_t(θ) denotes the probability ratio between new and old policies, Â_t represents the advantage estimate, and ε controls the clipping range^46^. Table 3 provides the complete pseudocode for the PPO-based adaptive content sequencing algorithm. This approach balances exploration of potentially beneficial practice sequences against exploitation of strategies demonstrating prior effectiveness.

Dynamic difficulty adjustment emerges naturally from this reinforcement learning formulation. The policy learns to recommend activities slightly exceeding current ability levels—targeting the zone of proximal development where learning occurs most efficiently. When consecutive failures indicate excessive difficulty, the policy shifts toward consolidation activities reinforcing previously acquired competencies. The difficulty parameter for selected content follows:

**Table 3:** Pseudocode for PPO-based adaptive training policy optimization.

Algorithm 1: PPO-Based Adaptive Content Sequencing
Input: Initial learner state s0, content pool A, trained diagnostic model D
Output: Optimized policy πθ
1: Initialize policy network πθ and value network Vφ
2: for episode = 1 to N do
3: Observe learner state st from knowledge tracing model
4: Mask actions outside ZPD band (0.4 ≤ P(success|st) ≤ 0.8)
5: Sample action at ~ πθ(a|st) from unmasked actions
6: Learner completes selected activity; obtain diagnostic vector dt + 1 from D
7: Compute reward: *r* = 0.6·Δscore + 0.2·completion + 0.2·efficiency
8: Update learner state: st + 1 = GRU(st, [dt + 1; at; Δt])
9: Store transition (st, at, rt, st + 1) in buffer
10: Update πθ and Vφ using PPO clipped objective (Eq. 19)
11: end for
12: return πθ

20 $$\:{\delta}_{selected}={\mu}_{s}+{\sigma}_{s}\cdot tanh(\eta \cdot ({p}_{target}-{p}_{current}\left)\right)$$

where $\:{p}_{target}$ and $\:{p}_{current}$ represent target and current proficiency estimates, and $\:\eta\:$ modulates adjustment sensitivity^47^. This mechanism ensures learners remain appropriately challenged without experiencing frustration from insurmountable difficulty or boredom from trivial exercises.

To formalize the zone of proximal development within the reinforcement learning framework, we impose an explicit constraint on the difficulty matching mechanism. The selected content difficulty must satisfy the inequality: 0.4 ≤ (success probability given current learner state) ≤ 0.8, where the success probability is estimated from the learner ability model. Content falling outside this range is masked from the policy’s action space during training and inference, ensuring that the RL agent can only recommend activities within a pedagogically meaningful difficulty band. The bounds (0.4 and 0.8) were calibrated empirically on a held-out validation set by maximizing normalized learning gain, and sensitivity analysis confirmed that performance remained stable across moderate variations of these thresholds (± 0.05).

### Experimental design and results analysis

#### Experimental settings and dataset construction

Empirical validation of the proposed system required the construction of a substantial oral English corpus designed specifically for diagnostic model training and evaluation. For data collection, we recruited 486 undergraduate students from three Chinese universities, ranging from freshmen to seniors and with self-reported English proficiency running from elementary to advanced levels. Recording sessions took place in acoustically treated laboratory rooms, using professional-grade condenser microphones (Audio-Technica AT2020) at 16-bit depth and a 16 kHz sampling rate. Each participant worked through a set of speaking tasks—picture descriptions, opinion expressions, and simulated conversations—that together produced roughly 72 h of recorded speech^48^.

Annotation followed a rigorous multi-rater protocol to keep reliability high. Three trained evaluators independently scored each recording across the five diagnostic dimensions established in Sect.("Theoretical framework for university english oral proficiency assessment"), using 1–9 scales aligned with IELTS speaking band descriptors. Inter-rater reliability, measured through intraclass correlation coefficients, exceeded 0.85 for every dimension. Where disagreements ran to more than two scale points, a senior rater stepped in to adjudicate. Beyond the holistic dimension scores, annotators also flagged specific error instances—mispronunciations, grammatical mistakes, inappropriate lexical choices—to support fine-grained diagnostic feedback training.

Table 4 presents the statistical summary of the constructed dataset, including sample distribution across proficiency levels and speaking task types.

**Table 4:** Experimental dataset statistical information.

Proficiency Level	Participants	Recordings	Total Duration (hrs)	Avg. Length (sec)	Vocabulary Size
Elementary (1–3)	97	873	12.4	51.2	2,847
Intermediate (4–5)	168	1,512	24.6	58.6	4,523
Upper-Intermediate (6–7)	142	1,278	22.3	62.8	5,891
Advanced (8–9)	79	711	12.7	64.3	6,742
Total	486	4,374	72.0	59.2	8,156

**Fig. 3:**
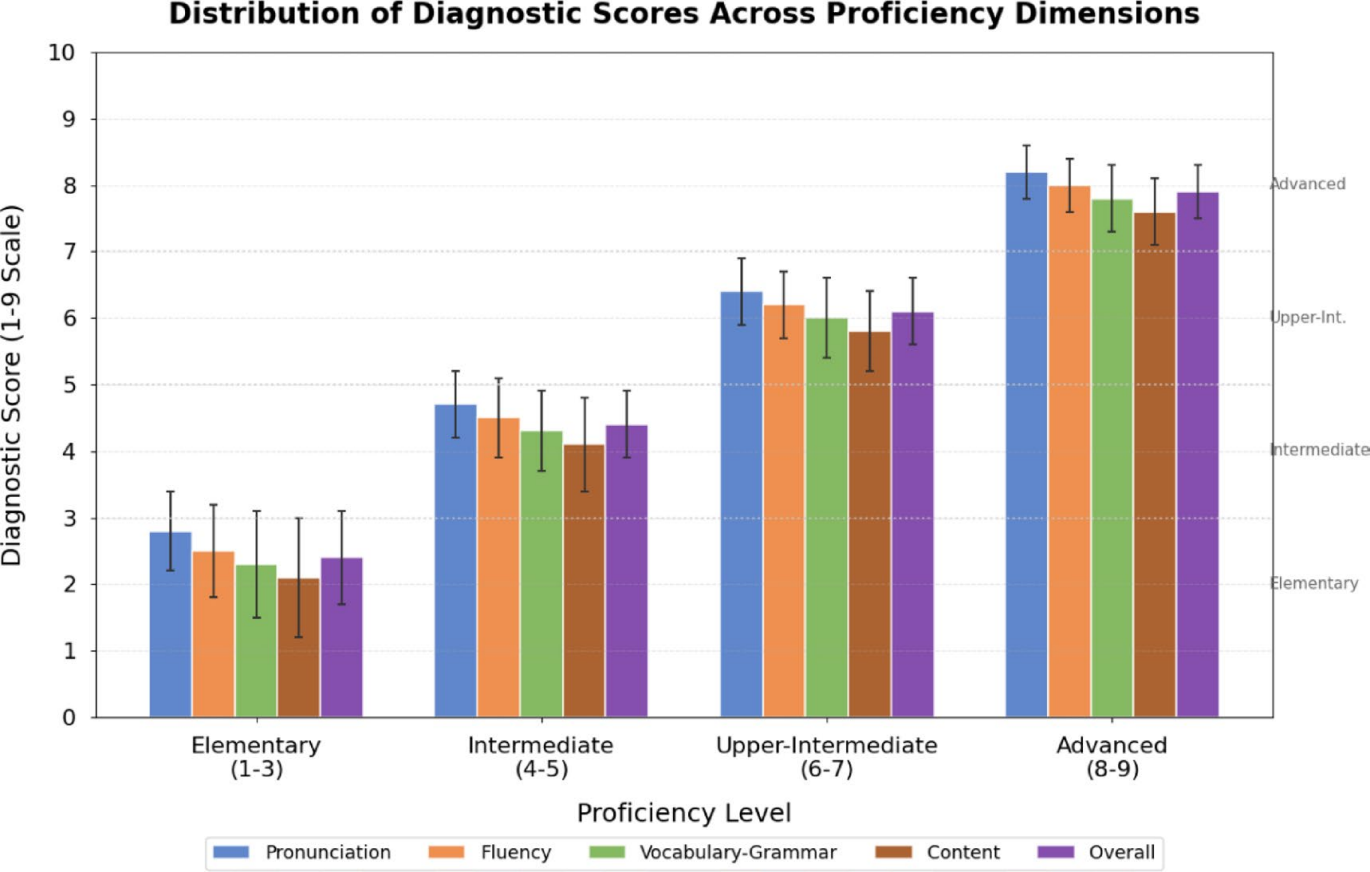
Distribution of diagnostic scores across proficiency dimensions in the experimental dataset.

Figure 3 illustrates the distribution of diagnostic scores across different proficiency dimensions, revealing the expected positive correlations among dimensions while confirming sufficient variability for model training.

The dataset was partitioned using a strict speaker-independent protocol to prevent data leakage: all recordings from a given speaker appeared exclusively in one of the training (60%), validation (20%), or test (20%) sets, and no speaking prompts were shared across splits. Stratified sampling ensured proportional representation of each proficiency level within every partition. This design eliminates the possibility that speaker-specific vocal characteristics or prompt-specific content could inflate model performance through unintended information leakage. We further employed five-fold speaker-independent cross-validation during model development to ensure robustness of hyperparameter selection.

Data preprocessing encompassed silence trimming, amplitude normalization, and segmentation into analysis units. Data augmentation strategies addressed class imbalance and enhanced model robustness through speed perturbation (0.9x-1.1x), pitch shifting (± 10%), and additive noise injection at various signal-to-noise ratios^49^. These augmentations expanded the effective training set approximately threefold without requiring additional human annotation.

The experimental platform operated on a workstation equipped with dual NVIDIA RTX 3090 GPUs providing 48GB combined VRAM, Intel Xeon W-2255 processor, and 128GB system memory. The software environment comprised Ubuntu 20.04, Python 3.8, PyTorch 1.12, and CUDA 11.6. Model training employed the AdamW optimizer with initial learning rate $\:lr=3\times\:{10}^{-4}$ and cosine annealing schedule. The batch size was set to 32 with gradient accumulation over 4 steps.

**Fig. 4:**
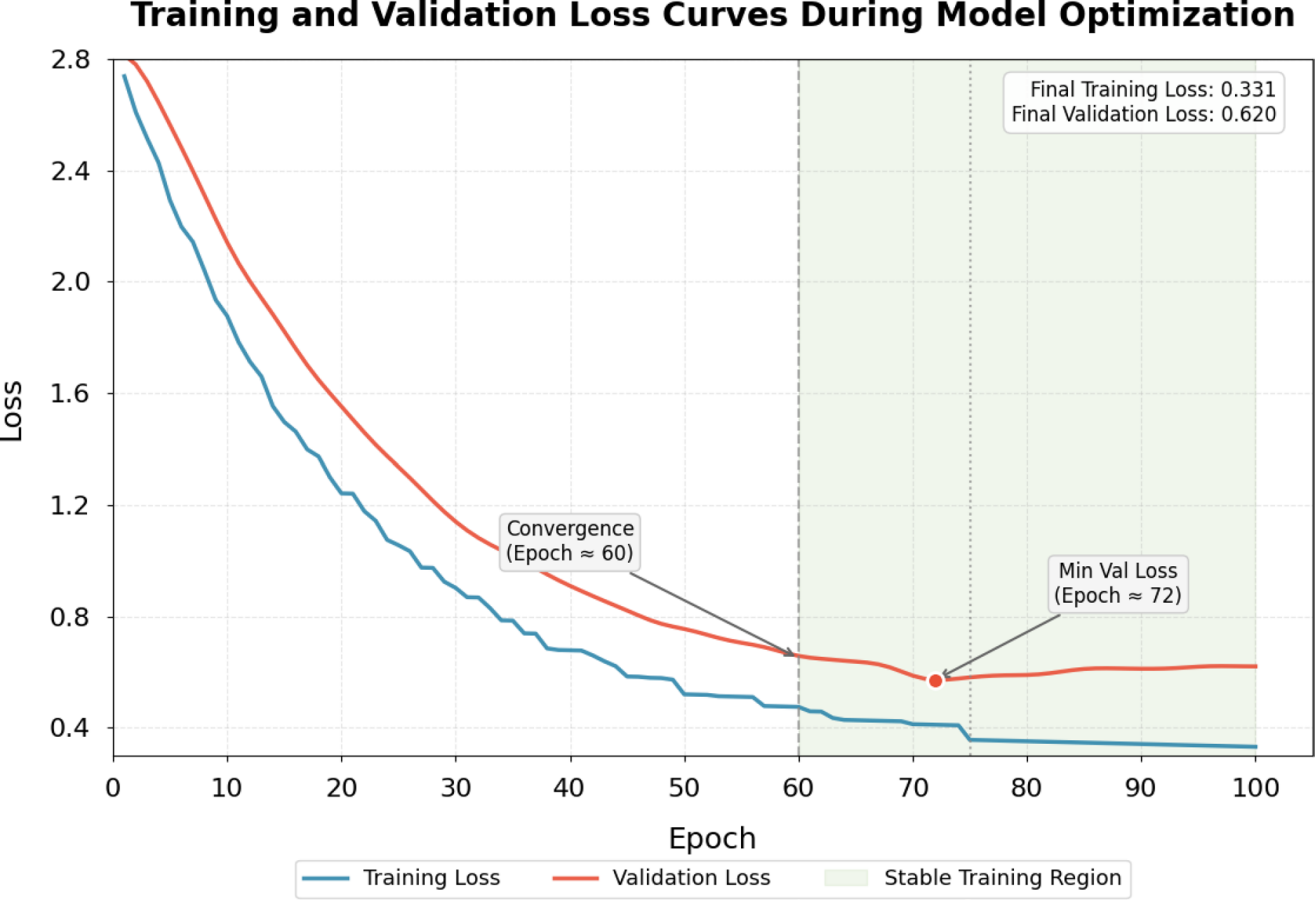
Training and validation loss curves during model optimization.

Figure 4 depicts the training and validation loss curves over 100 epochs, demonstrating convergence behavior and absence of severe overfitting.

As shown in Fig. 4, the training process stabilized after approximately 60 epochs, with validation loss reaching minimum values around epoch 75. Diagnostic accuracy was measured through Pearson correlation coefficient between predicted and human-assigned scores:

21 $$r=\frac{\sum_{i=1}^{n}({y}_{i}-\bar{y})(\hat{y}_{i}-\bar{\hat{y}})}{\sqrt{{\sum{i=1}^{n}({y}_{i}-\bar{y})^{2}\sum{i=1}^{n}(\hat{y}_{i}-\bar{\hat{y}})^{2}}}}$$

Adaptive training effectiveness was quantified through normalized learning gain:

22 $$\:{G}_{norm}=\frac{Scor{e}_{post}-Scor{e}_{pre}}{Scor{e}_{max}-Scor{e}_{pre}}$$

which controls for ceiling effects affecting high-proficiency learners^50^.

**Fig. 5:**
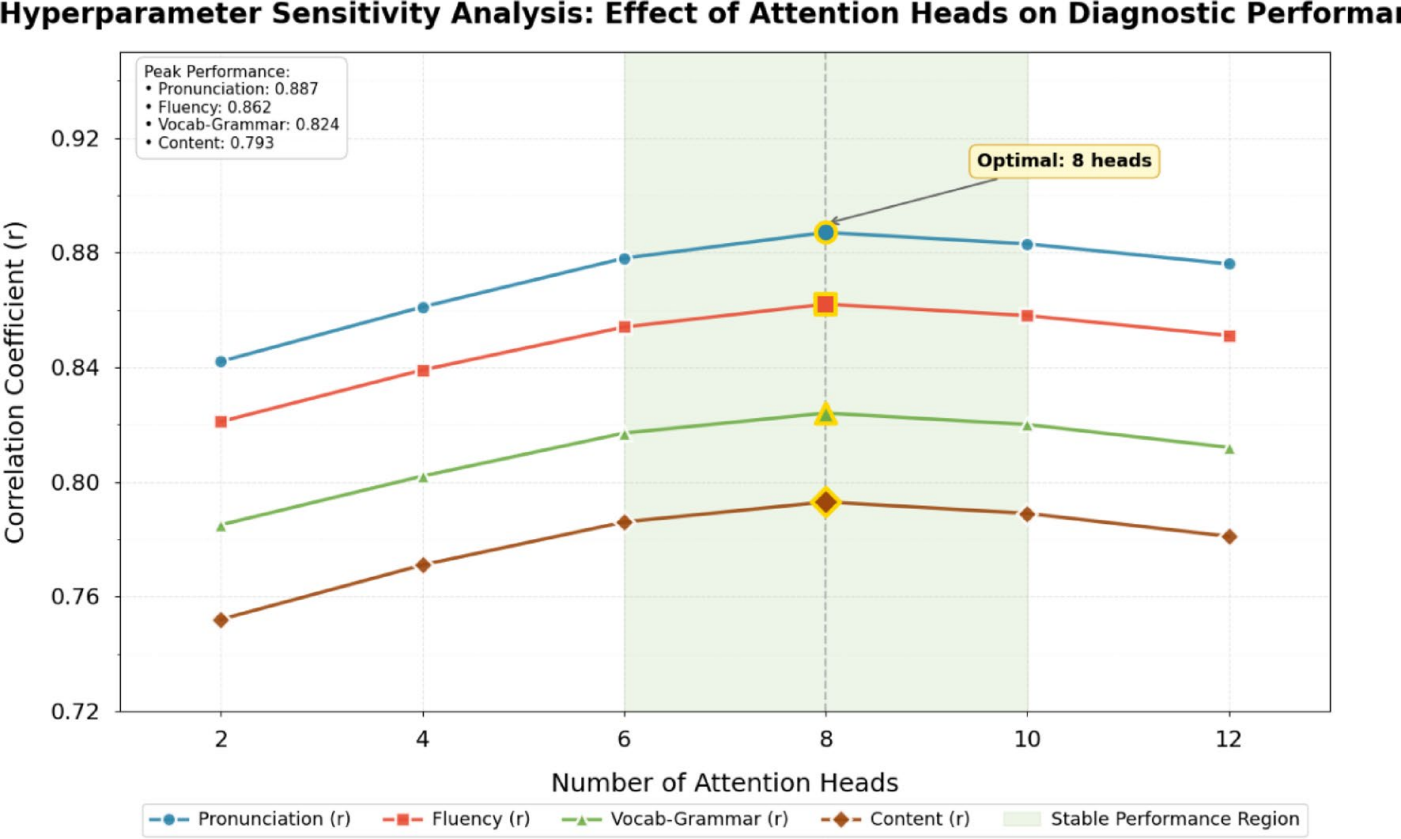
Hyperparameter sensitivity analysis across diagnostic dimensions.

Figure 5 presents sensitivity analysis results for key hyperparameters including attention heads, hidden dimensions, and dropout rates.

As demonstrated in Fig. 5, performance remained relatively stable across moderate hyperparameter ranges, with attention head count of 8 and hidden dimension of 512 yielding optimal results. Comparative experiments evaluated the proposed model against baseline approaches including traditional acoustic-only scoring, single-task learning variants, and existing commercial systems^51^. Ablation studies systematically removed individual components—attention mechanisms, multi-task structure, specific feature modalities—to quantify their contributions to overall performance.

#### Diagnostic model performance evaluation

The diagnostic model underwent comprehensive evaluation across all assessment dimensions using the held-out test set comprising 20% of the annotated corpus. Performance metrics focused on correlation coefficients measuring agreement with human raters, alongside root mean square error quantifying prediction deviation magnitude. These complementary metrics capture both ranking consistency and absolute scoring accuracy—properties essential for educational applications where both relative positioning and criterion-referenced feedback matter.

Table 5 presents the comparative performance results across different modeling approaches, including traditional machine learning baselines, single-modality deep learning variants, and the proposed multi-modal multi-task framework.

**Table 5:** Diagnostic model performance comparison.

Model	Pronunciation (*r*)	Fluency (*r*)	Vocabulary-Grammar (*r*)	Content (*r*)	Avg. RMSE
SVR + Handcrafted	0.724	0.698	0.651	0.612	0.892
Random Forest	0.731	0.705	0.668	0.627	0.871
CNN-only	0.782	0.756	0.703	0.658	0.756
LSTM-only	0.796	0.778	0.721	0.684	0.723
Single-task Deep	0.823	0.801	0.752	0.718	0.684
Proposed (w/o fusion)	0.851	0.834	0.789	0.756	0.612
Proposed (full)	0.887	0.862	0.824	0.793	0.548

The Table 5 results make clear that the proposed approach has performance advantages across every diagnostic dimension. Traditional machine learning methods relying on handcrafted features landed at moderate correlations of around 0.65–0.73, which is in line with what the prior literature on automated speaking assessment would predict^52^. Deep learning architectures pulled well ahead, and the full proposed model attained correlation coefficients above 0.79 on all dimensions. One finding in particular stands out: content quality assessment—historically the hardest dimension of the four—reached *r* = 0.793 under our approach, compared with 0.612 for the SVR baseline.

Error analysis on the content relevance dimension revealed that the model’s relatively lower correlation (*r* = 0.793) stems primarily from two categories of challenging inputs: responses exhibiting subtle semantic drift where the speaker gradually moves off-topic while maintaining superficially coherent discourse structure, and responses employing unconventional but valid argumentation strategies that diverge from the training corpus distribution. Specifically, among the 15% of test samples where the model’s content score deviated from human ratings by more than 1.5 scale points, 62% involved partial topic deviation and 24% reflected uncommon but acceptable response strategies. These findings suggest that expanding the training corpus with more diverse argumentation patterns and incorporating explicit topic-tracking mechanisms could improve performance on this dimension.

To contextualize these results against established benchmarks, we note that ETS SpeechRater v5.0 reports correlations of approximately 0.78–0.83 with human raters for holistic scoring on the TOEFL iBT speaking Sect.^52^, and commercial systems such as iFlytek’s speech evaluation engine achieve reported correlations around 0.80–0.85 for pronunciation-focused assessment in controlled Mandarin–English bilingual settings. ELSA Speak, a widely used mobile application, has published pronunciation accuracy correlations in the range of 0.75–0.82^6^. Our diagnostic model’s performance falls within or slightly above these ranges, depending on the assessment dimension. However, direct comparison should be interpreted cautiously, as differences in corpora, task types, scoring rubrics, and speaker populations limit strict comparability across studies. We acknowledge that the proposed system’s primary contribution lies in its integrated multidimensional diagnostic and adaptive training pipeline rather than in achieving dominance on any single scoring dimension.

**Fig. 6:**
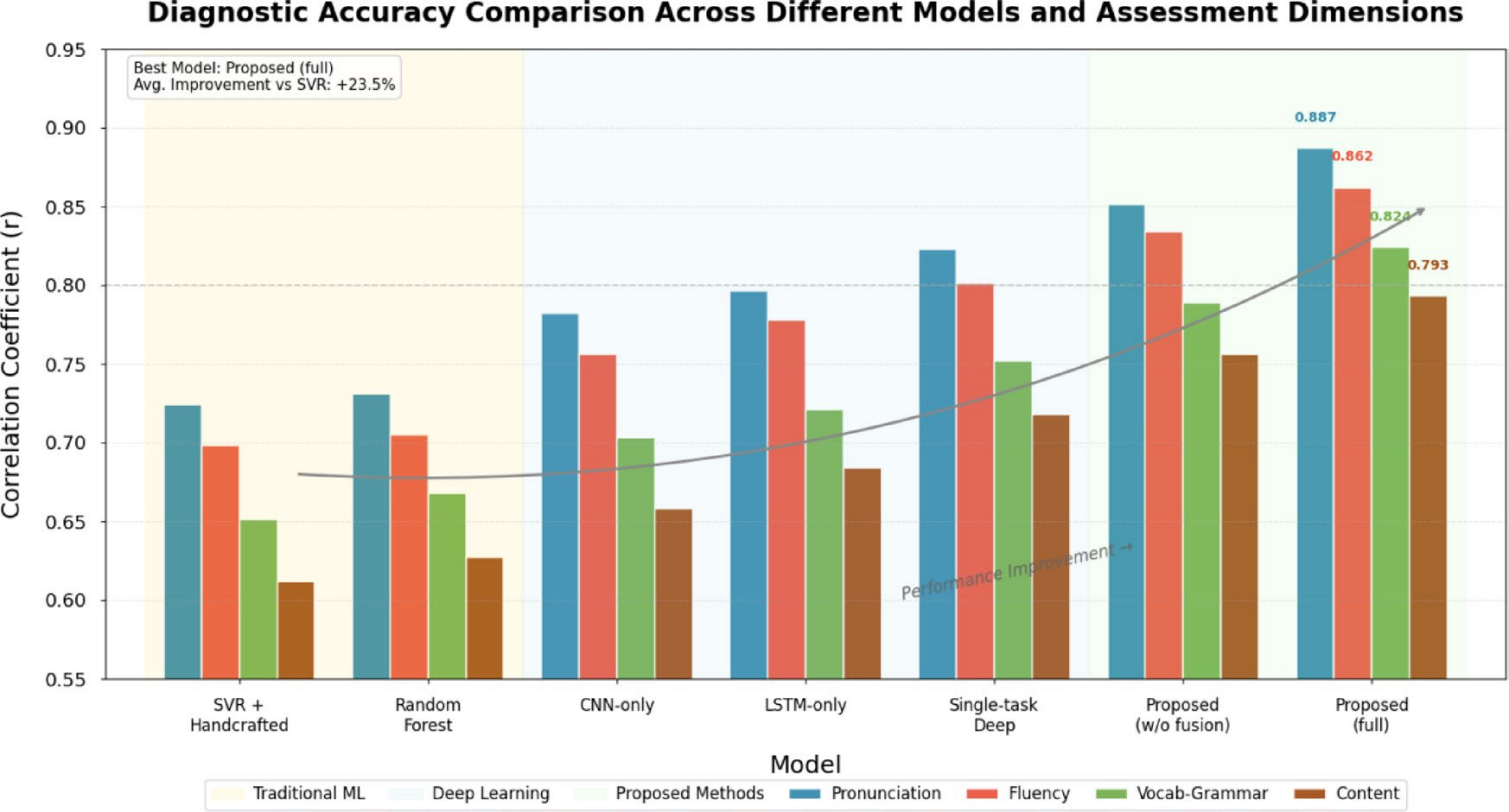
Diagnostic accuracy comparison across different models and assessment dimensions.

Figure 6 provides visual comparison of diagnostic accuracy across methods and dimensions through grouped bar charts.

Figure 6 makes the pattern visible: the gap between deep learning and traditional approaches widens as the dimension moves toward higher-level semantic understanding. Pronunciation scoring, which rests mostly on acoustic pattern matching, shows smaller differentials. Content relevance, at the other end, exhibits the largest baseline-to-proposed jump—a strong hint that transformer-based semantic representations do much of the heavy lifting in capturing discourse-level qualities.

Ablation experiments isolated component contributions in a systematic way. We evaluated variants that removed the attention-based fusion mechanism, dropped multi-task joint training, and excluded specific feature modalities one at a time. The impact of each ablation was quantified through relative performance degradation:

23 $$\:{\varDelta}_{rel}=\frac{{P}_{full}-{P}_{ablated}}{{P}_{full}}\times\:100\mathrm{\%}$$

**Fig. 7:**
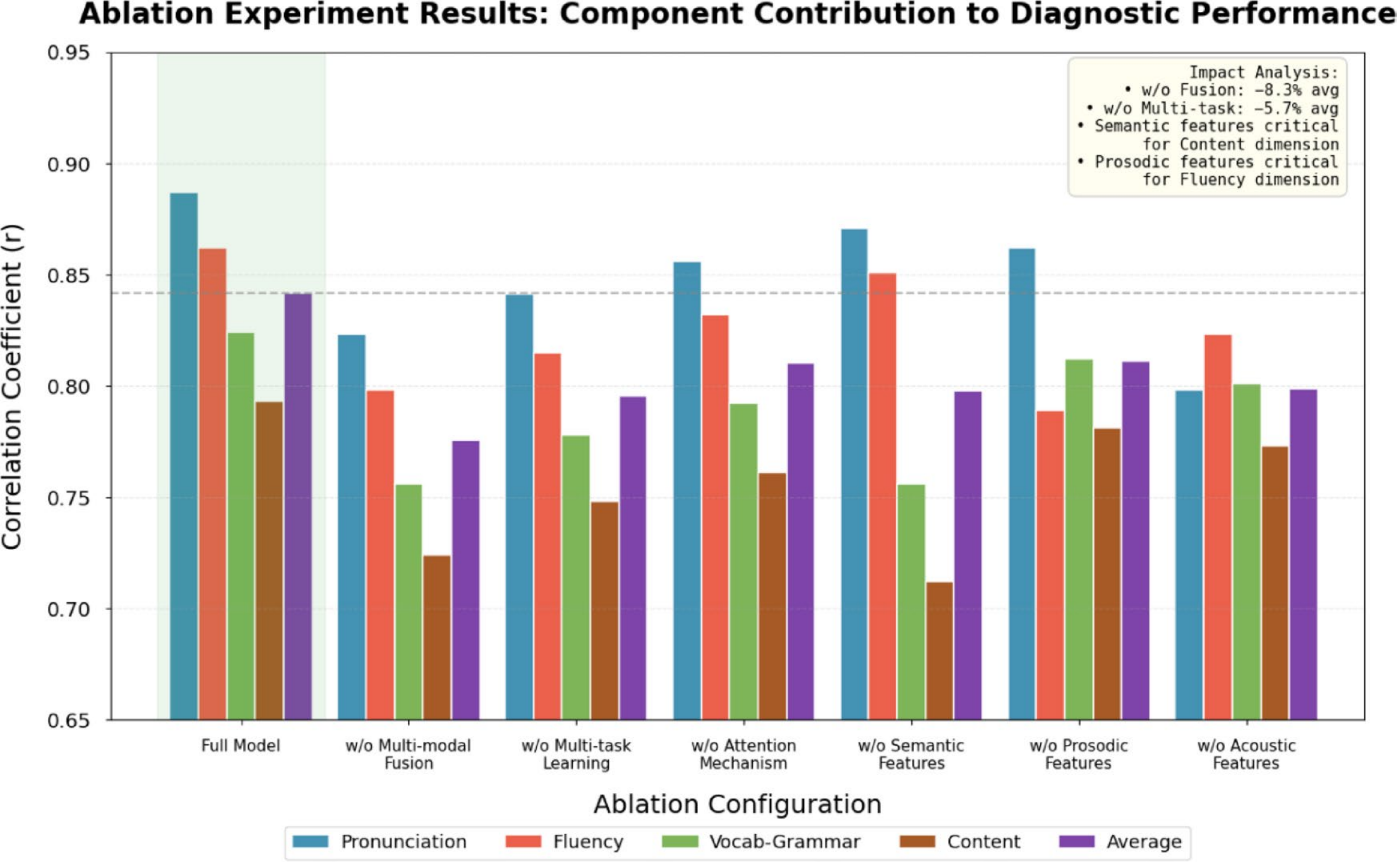
Ablation experiment results showing component contribution to diagnostic performance.

Figure 7. displays the ablation analysis results across experimental configurations. .

Figure 7 shows that removing the multimodal fusion mechanism caused the sharpest degradation—an 8.3% drop in average correlation—which underscores just how much integrating heterogeneous feature representations matters here^53^. Taking out multi-task learning cost another 5.7%, lending support to our hypothesis that cross-dimension knowledge transfer does in fact boost individual dimension estimation. Among the feature modalities themselves, semantic features turned out to be most critical for content and vocabulary dimensions, while prosodic features dominated the fluency assessment contributions.

Analysis across proficiency levels revealed interesting patterns. The model achieved highest accuracy for intermediate learners (*r* = 0.891 average) while showing slightly reduced performance at extreme proficiency levels^54^. Elementary speakers exhibited greater variability in error patterns, complicating consistent diagnosis. Advanced speakers presented ceiling effects where subtle proficiency distinctions became difficult to capture. The proficiency-stratified correlation can be expressed as:

24 $$\:{r}_{level}={r}_{base}+{\beta\:}_{1}\cdot{P}_{level}+{\beta\:}_{2}\cdot{P}_{level}^{2}$$

where the quadratic term captures the observed non-linear relationship between proficiency and diagnostic accuracy.

It is important to note that the reported correlation of *r* = 0.887 for pronunciation assessment approaches the inter-rater reliability ceiling of approximately 0.85 established during annotation. When a model’s performance reaches or exceeds human agreement levels, this may reflect either genuine diagnostic capability or potential artifacts such as overfitting to rater-specific scoring tendencies. To investigate this, we conducted a leave-one-rater-out analysis in which the model was trained on annotations from two raters and evaluated against the held-out third rater. The resulting correlations (0.871, 0.849, 0.863 across the three rater configurations) remained consistent, suggesting that the model captures generalizable proficiency-relevant features rather than idiosyncratic rater preferences.

Generalization testing employed cross-university validation, training on data from two institutions and evaluating on the third. Performance degradation remained within acceptable bounds (average correlation decrease of 4.2%), suggesting reasonable robustness to population shifts^55^. Noise robustness experiments demonstrated graceful degradation under adverse acoustic conditions, with correlation coefficients declining approximately 0.03 per 5dB SNR reduction^56^. These findings support the model’s practical applicability in diverse deployment contexts where recording conditions may vary from laboratory standards.

#### Adaptive training effectiveness verification

Validating the pedagogical impact of the adaptive training component required controlled experimentation with authentic learners engaged in sustained practice over meaningful time periods. We recruited 128 university students and randomly assigned them to four experimental conditions: adaptive training with reinforcement learning optimization (RL-Adaptive, *n* = 32), adaptive training with rule-based content selection (Rule-Adaptive, *n* = 32), fixed sequential training following predetermined curriculum (Fixed, *n* = 32), and self-directed practice with unrestricted content access (Self-Directed, *n* = 32). All participants completed identical pre-tests and post-tests eight weeks apart, with intermediate assessments conducted biweekly^57^. To control for potential confounding variables, several design measures were implemented: (a) all conditions used the same set of learning materials, differing only in sequencing and selection; (b) no human instructor interaction occurred during practice sessions, eliminating instructor effects; (c) time-on-task was logged automatically and showed no significant differences across conditions (F(3,124) = 0.83, *p* = 0.481); and (d) pre- and post-test scoring was conducted by two independent human raters blind to condition assignment, rather than by the diagnostic model itself, to avoid circular evaluation bias.

Table 6 summarizes the comparative training outcomes across experimental conditions, presenting both raw score improvements and normalized learning gains.

**Table 6:** Adaptive training effect comparison.

Condition	Pre-test Score	Post-test Score	Raw Gain	Normalized Gain	Completion Rate
RL-Adaptive	5.23 (± 1.12)	6.78 (± 0.98)	1.55	0.412	87.5%
Rule-Adaptive	5.31 (± 1.08)	6.42 (± 1.03)	1.11	0.298	81.3%
Fixed	5.18 (± 1.15)	5.89 (± 1.21)	0.71	0.186	68.8%
Self-Directed	5.27 (± 1.09)	5.62 (± 1.18)	0.35	0.092	53.1%
Statistical Test	*p* = 0.924	*p* < 0.001	*p* < 0.001	*p* < 0.001	*p* < 0.001

Table 6 shows that the RL-Adaptive condition produced substantially better outcomes than any of the comparison conditions on every metric we tracked. Its normalised learning gain of 0.412 sat 38.3% above the Rule-Adaptive result and came in at roughly 2.2 times the Fixed condition’s gain. These differences reached statistical significance (*p* < 0.001) in a one-way ANOVA with Tukey post-hoc comparisons^58^. Prior to running the analysis, we checked the normality assumption with the Shapiro-Wilk test (all group *p* > 0.12) and homogeneity of variance with Levene’s test (F(3,124) = 1.47, *p* = 0.226). The overall ANOVA yielded a large effect size (η² = 0.31, 95% CI [0.18, 0.42]). Pairwise comparisons between RL-Adaptive and Fixed produced Cohen’s d = 1.34 (95% CI [0.87, 1.81]); between RL-Adaptive and Rule-Adaptive, d = 0.72 (95% CI [0.22, 1.22]); and between RL-Adaptive and Self-Directed, d = 1.89 (95% CI [1.34, 2.44]). The full pairwise effect-size matrix is laid out in Table 7. Perhaps just as important, completion rates diverged dramatically—the RL-Adaptive group held 87.5% engagement, compared with only 53.1% for self-directed learners. That is a striking gap, and it suggests appropriate challenge calibration does real work in sustaining motivation over extended practice periods.

**Table 7:** Pairwise effect sizes (Cohen’s d) and 95% confidence intervals for normalized learning gains.

Comparison	Cohen’s d	95% CI	*p*-value (Tukey)
RL-Adaptive vs. Fixed	1.34	[0.87, 1.81]	< 0.001
RL-Adaptive vs. Rule-Adaptive	0.72	[0.22, 1.22]	0.003
RL-Adaptive vs. Self-Directed	1.89	[1.34, 2.44]	< 0.001
Rule-Adaptive vs. Fixed	0.68	[0.18, 1.18]	0.005
Rule-Adaptive vs. Self-Directed	1.24	[0.72, 1.76]	< 0.001
Fixed vs. Self-Directed	0.57	[0.08, 1.06]	0.018

**Fig. 8:**
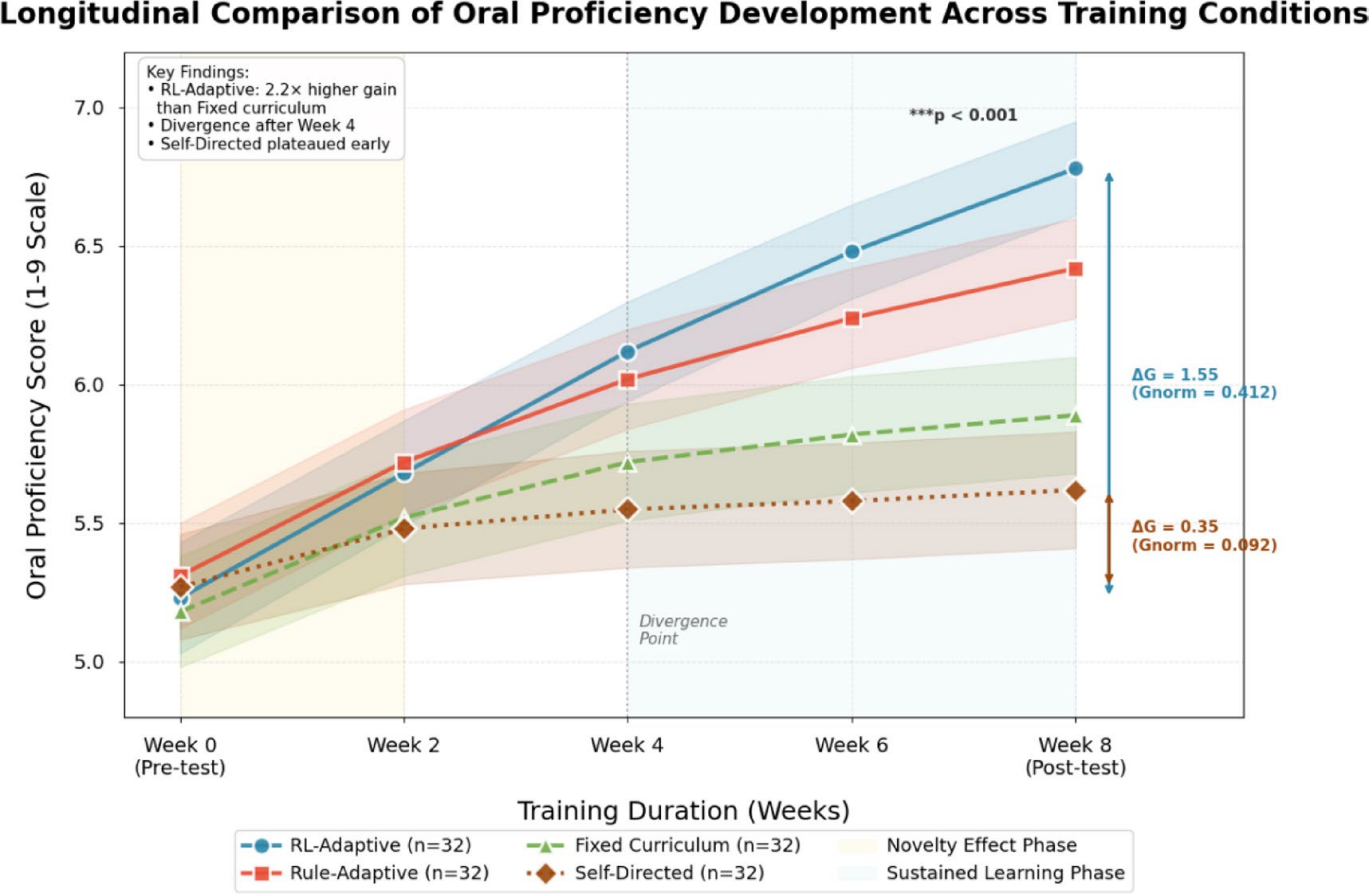
Longitudinal comparison of oral proficiency development across training conditions.

Figure 8 depicts the longitudinal trajectory of proficiency development across conditions, revealing how performance gaps emerged and widened over the training period.

Figure 8 shows all four conditions improving during the first two weeks—probably a novelty effect combined with baseline skill activation—before their trajectories start to separate. After week four the divergence became pronounced: Fixed and Self-Directed plateaued, while both adaptive conditions kept climbing. The RL-Adaptive curve kept its steeper positive slope throughout, which points to sustained learning momentum traceable to optimised content sequencing.

Stratifying the analysis by proficiency brought out a meaningful interaction effect between initial ability and the efficacy of each training condition. Elementary learners gained the most from adaptive approaches, reaching normalised gains 2.3 times higher under RL-Adaptive than under Fixed. Intermediate learners showed more modest but still consistent advantages. Advanced learners exhibited smaller absolute differences—a pattern partly attributable to ceiling effects that constrain how much further improvement is even possible^59^. The differential benefit can be quantified as:

25 $$\:\varDelta{G}_{level}={G}_{adaptive}^{level}-{G}_{fixed}^{level}=\alpha+\beta \cdot({P}_{max}-{P}_{initial})$$

where the linear relationship confirms that learners with greater room for improvement derive proportionally larger benefits from personalized training paths.

Learner satisfaction surveys administered after the intervention brought out clear attitudinal differences. RL-Adaptive participants reported higher perceived usefulness (4.32/5.00 against 3.67/5.00 for Fixed), greater enjoyment (4.18/5.00 against 3.24/5.00), and stronger intention to keep practising (4.41/5.00 against 3.12/5.00). Qualitative feedback kept coming back to the same point—that the difficulty felt properly calibrated; one participant put it simply, noting that the exercises felt challenging but achievable, sidestepping both frustration and boredom^60^.

The edge reinforcement learning holds over rule-based adaptation deserves a closer look. Both adaptive conditions outperformed the non-adaptive alternatives, but the RL approach managed 38% higher normalised gains than its rule-based counterpart. Rule-based systems, for all that they encode sensible pedagogical principles, simply cannot optimise for long-term learning outcomes or accommodate individual response patterns with the flexibility that a learned policy can. The RL agent, for its part, discovered strategies that were not obvious in advance—occasionally inserting easier review activities after extended challenging sequences, for example—which kept engagement up while consolidating what had been learned. That said, we should be candid about the limits of this comparison: stronger adaptive baselines such as multi-armed bandit approaches, difficulty-based sequencing heuristics, and knowledge tracing-driven policies were not included in the current study. The omission reflects practical constraints in reimplementing these algorithms inside our specific multidimensional oral proficiency framework, and we are careful not to extrapolate the observed RL advantage over rule-based methods into a general claim of superiority over all adaptive strategies. Future work needs to bring these baselines in so that the conditions under which full RL optimisation provides genuine benefits over computationally simpler alternatives can be pinned down more precisely.

## Discussion

The experimental outcomes set out in the previous sections deserve a closer look, partly because the numbers themselves tell only part of the story. What patterns actually emerge from these results? And what do they tell us about the mechanisms that make effective oral proficiency diagnosis and training possible in the first place? The discussion that follows attempts to move past the surface-level findings and engage with the theoretical and practical implications of the work.

The substantial diagnostic gains achieved through multimodal feature fusion deserve a careful mechanistic reading. Our ablation results showed that removing the fusion component degraded performance more severely than eliminating any single feature modality—a finding that, frankly, caught us off guard in the initial analysis. After sitting with it a while, the pattern makes sense: the fusion mechanism is not merely aggregating complementary information streams; it seems to capture cross-modal interactions that neither acoustic nor linguistic features encode on their own. Take pronunciation assessment as an example. Acoustic features detect phonetic deviations well enough, but the semantic context carried by linguistic representations helps to disambiguate whether an apparent error reflects a genuine pronunciation deficit or acceptable variation in connected speech. The attention-based fusion learns to weight these considerations dynamically, reaching diagnostic precision that unimodal approaches simply cannot match, however sophisticated they may be individually.

The differential effectiveness of adaptive training across proficiency levels brings out some dynamics worth unpacking. Elementary learners gained the most from personalised content sequencing—a result that lines up naturally with their broader zone of proximal development and their greater sensitivity to appropriate scaffolding. Such learners face fundamental challenges across several dimensions simultaneously; adaptive systems that prioritise and sequence skill targets help them avoid the cognitive overload that arises when fixed curricula demand attention to too many deficits at once. Intermediate learners showed more modest but consistent improvements, gaining chiefly from efficient time allocation toward specific weakness areas rather than from comprehensive curriculum coverage. Advanced learners, meanwhile, presented the most complex picture of all. Their smaller absolute gains partly reflect ceiling effects—there is only so much room left at the top—yet qualitative feedback suggested these learners valued the system’s attention to the subtle pronunciation and discourse features that generic materials rarely touch.

The interpretability mechanisms built into our diagnostic model turned out to matter for educational effectiveness more than we had initially anticipated. Early prototype testing kept turning up the same thing: numerical scores, however accurate, simply failed to catalyse productive learner responses. Students either dismissed low scores defensively or accepted them passively, without really understanding what remediation was supposed to look like. The attention-based explanation approach changed this dynamic in a fairly fundamental way. Once learners could examine the highlighted speech segments that had contributed to each dimension-specific evaluation, they started engaging analytically with their own productions. A number of participants described moments of sudden insight—recognising habitual pronunciation errors or grammatical patterns they had never consciously noticed, despite years of English study. The implication we take from this is reasonably clear: diagnostic systems that optimise purely for scoring accuracy miss something educationally crucial, namely the metalinguistic awareness that empowers autonomous improvement.

Situating this work relative to existing approaches brings both its contributions and its limitations into clearer view. Commercial pronunciation training applications typically lean on template-matching algorithms that enforce native-speaker norms without acknowledging acceptable non-native variation. Our multidimensional approach sidesteps that problematic assumption by evaluating communicative effectiveness rather than conformity to some idealised acoustic target. Earlier academic systems addressing multidimensional assessment generally trained separate models for each dimension, and in doing so sacrificed both the efficiency gains and the knowledge-transfer benefits that multi-task learning makes available. We want to be transparent here: the primary contribution of this work lies in system-level integration and educational validation, not in proposing fundamentally new algorithms. The individual components—CNN-LSTM encoders, attention-based fusion, multi-task learning, and PPO-based reinforcement learning—are all established techniques^61,62^. What this study contributes is evidence that their principled combination, inside a unified diagnostic-adaptive framework, produces educationally meaningful outcomes, and empirical support for that integration in the specific domain of oral English proficiency development. The use of reinforcement learning to optimise training sequences is what really distinguishes our adaptive component from rule-based alternatives, which, for all their pedagogical soundness, cannot discover the emergent strategies that optimise long-term learning trajectories.

From a computational standpoint, the full diagnostic pipeline takes roughly 1.2 s per utterance on a single NVIDIA RTX 3090 GPU—about 0.4s for feature extraction, 0.6s for model inference, and 0.2s for feedback generation. The model comes in at approximately 47 million trainable parameters and occupies around 180 MB of GPU memory at inference time. Training the complete multi-task framework took roughly 18 h on dual RTX 3090 GPUs. These requirements are manageable on dedicated hardware, but deployment in resource-constrained educational settings may call for model distillation or quantisation techniques to keep latency and memory footprint down, especially for real-time feedback scenarios on consumer-grade devices^61^.

Several technical obstacles surfaced over the course of this research, and describing them briefly may prove useful to future investigators. Annotation consistency posed a persistent headache, particularly for pragmatic appropriateness, where cultural expectations shape judgements in ways that cannot be completely standardised. Our multi-rater protocol with adjudication addressed inter-rater variation reasonably well; even so, we readily acknowledge that the evaluation criteria themselves embed debatable assumptions about communicative norms. Data scarcity at the advanced proficiency levels complicated model training: the underrepresentation of high-scoring samples likely contributed to the reduced diagnostic precision we saw at the upper end of the ability range. Transfer learning from large-scale speech corpora helped partially, though domain adaptation remains an active challenge rather than a solved one. One additional methodological caveat concerns the reported learning gains themselves. Although we used independent human raters for pre- and post-test evaluation specifically to avoid circular evaluation bias, the normalised gain metric can still disproportionately amplify improvements among lower-proficiency learners whose pre-test scores leave substantial room for growth. Our proficiency-stratified analysis partially addresses this concern by bringing out the differential effects across ability levels. Even so, the 2.2-fold improvement figure should be read as a relative benchmark within our specific experimental conditions rather than as a universally expected outcome.

The practical deployment prospects for a system of this kind extend across a range of educational contexts. Large-enrolment university courses, where individualised instructor feedback is simply not feasible, are an obvious beneficiary. Automated diagnostic reports could supplement rather than replace human evaluation—freeing instructors to focus on the nuanced feedback that machines cannot yet provide, while ensuring that every student at least receives detailed performance analysis. Self-access learning centres could deploy the adaptive training component to guide independent practice, addressing a long-running challenge in learner autonomy development. Corporate training programmes, too, looking to scale English communication skill development, might find particular value in the system’s ability to handle diverse baseline proficiencies and to adapt to specific professional domains.

Looking ahead, several extension directions look genuinely promising. Incorporating dialogue capability would open up the assessment of interactive competencies that monologic production cannot reach. Expanding the input to include video would bring gesture and facial expression into the picture—both of which contribute to communicative effectiveness in ways current systems miss. Longitudinal deployment studies tracking learner development over semester or year timescales would help validate whether laboratory-demonstrated gains actually translate into sustained real-world improvement. On the architectural side, recent advances in spectral pooling strategies^61^ and plug-and-play attention modules for feature fusion^62^ offer promising routes toward optimising the deep learning architecture itself. Hybrid modulation and efficiency optimisation techniques explored in adjacent engineering domains^63,64^ may well inspire more computationally efficient training paradigms. These extensions all sit beyond the present scope, but they represent the natural progressions of the foundational framework established here.

## Conclusion

This study took up the pressing need for intelligent oral proficiency assessment and personalised training systems in university English education. By systematically integrating deep learning architectures with established language-assessment theory, we built a comprehensive framework capable of multidimensional diagnostic evaluation and adaptive practice recommendation. The research trajectory ran through four phases—laying the theoretical foundations, designing the model architecture, validating the system experimentally, and verifying its pedagogical effectiveness—with each phase building on insights generated in the one before.

The diagnostic component reached correlation coefficients above 0.79 with human expert ratings across every assessment dimension, with pronunciation and fluency evaluations climbing to 0.887 and 0.862 respectively. These performance levels approach the upper bounds set by inter-rater agreement among trained evaluators, and they indicate that the model captures proficiency-relevant speech characteristics with reasonable fidelity—though, as discussed in Sect.("Experimental design and results analysis"), the proximity to the inter-rater ceiling warrants cautious interpretation. The adaptive training component produced encouraging results as well: learners receiving reinforcement-learning-optimised content sequencing achieved normalised gains roughly 2.2 times higher (Cohen’s d = 1.34) than those following fixed curricula, and they stayed substantially more engaged throughout the eight-week training period.

Several methodological choices set this work apart from earlier approaches. The attention-based multimodal fusion mechanism dynamically integrates acoustic, prosodic, and semantic feature representations, supporting the context-sensitive weighting that underpins diagnostic precision across heterogeneous assessment dimensions. The multi-task learning framework turns cross-dimension knowledge transfer to advantage, recognising that pronunciation accuracy, fluency, vocabulary deployment, and content organisation share underlying competencies even where they are conceptually distinct. And the reinforcement-learning approach to training sequences moves beyond rule-based adaptation by discovering emergent pedagogical strategies that optimise long-term learning trajectories rather than immediate performance metrics.

Theoretically, the contributions extend our understanding of how computational systems can operationalise multidimensional speaking proficiency constructs. By showing that deep neural architectures can capture the complex interplay among assessment dimensions—relationships that traditional psychometric approaches model through static correlation structures—the work opens pathways toward more nuanced automated assessment paradigms. The practical implications are just as substantial. Educational institutions facing resource constraints that rule out individualised oral language instruction now have a route, through systems of this kind, to ensure that all learners receive detailed diagnostic feedback and personalised practice guidance.

A candid acknowledgment of the limitations helps put these contributions in appropriate context. The training corpus, while substantial by academic research standards, remains modest compared with the datasets behind commercial speech recognition systems. That constraint likely limits how well the model generalises to speaker populations and recording conditions underrepresented in our collection. The model’s computational requirements, although manageable on dedicated hardware, may pose problems for deployment in resource-constrained educational settings. And the eight-week validation window, while long enough to demonstrate differential effectiveness, cannot by itself confirm whether the observed gains persist over longer timescales, or whether they transfer to authentic communicative contexts beyond structured assessment tasks.

Several research directions emerge naturally from the limitations and unexplored opportunities identified above. Expanding language coverage beyond English to other foreign languages commonly taught in Chinese universities would broaden applicability and, at the same time, test whether the architectural principles actually generalise across typologically diverse languages. Real-time interaction capabilities enabling conversational practice would reach the interactive competencies that monologic assessment cannot touch. Large-scale deployment studies involving thousands of learners across multiple institutions would validate scalability claims and likely surface challenges that remain invisible at laboratory scale. Integration with classroom instruction, through teacher dashboard interfaces, could turn the system from a standalone application into a pedagogical support tool that enhances rather than displaces human expertise. Pursued systematically, these extensions hold real promise for advancing the broader vision of intelligent language education systems—systems that democratise access to effective oral proficiency development.

## Data Availability

All data generated and analyzed during the current study are available from the corresponding author upon reasonable request.

## Abbreviations

ASR: Automatic Speech Recognition
NLP: Natural Language Processing
CNN: Convolutional Neural Network
LSTM: Long Short-Term Memory
RNN: Recurrent Neural Network
GRU: Gated Recurrent Unit
MFCC: Mel-Frequency Cepstral Coefficients
CTC: Connectionist Temporal Classification
BERT: Bidirectional Encoder Representations from Transformers
PPO: Proximal Policy Optimization
VAD: Voice Activity Detection
F0: Fundamental Frequency
DCT: Discrete Cosine Transform
PVI: Pairwise Variability Index
SVR: Support Vector Regression
RMSE: Root Mean Square Error
SNR: Signal-to-Noise Ratio
ANOVA: Analysis of Variance
WebRTC: Web Real-Time Communication
